# Molecular Mechanisms and Clinical Aspects of Colitis-Associated Cancer in Ulcerative Colitis

**DOI:** 10.3390/cells14030162

**Published:** 2025-01-22

**Authors:** Jesus K. Yamamoto-Furusho, Fausto D. Gutierrez-Herrera

**Affiliations:** Inflammatory Bowel Disease Clinic, Department of Gastroenterology, Instituto Nacional de Ciencias Medicas y Nutricion Salvador Zubiran, Ciudad de México 14080, Mexico; fausto.gutierrez7859@alumnos.udg.mx

**Keywords:** ulcerative colitis, dysplasia, molecular mechanisms, colitis-associated cancer

## Abstract

Inflammatory bowel diseases have long been recognized as entities with a higher risk of colorectal cancer. An increasing amount of information has been published regarding ulcerative colitis-associated colorectal cancer and its unique mechanisms in recent decades, as ulcerative colitis constitutes a chronic process characterized by cycles of activity and remission of unpredictable durations and intensities; cumulative genomic alterations occur during active disease and mucosal healing, resulting in a special sequence of events different to the events associated with sporadic colorectal cancer. The recognition of the core differences between sporadic colorectal cancer and colitis-associated cancer is of great importance to understand and guide the directions in which new research could be performed, and how it could be applied to current clinical scenarios. A DSS/AOM murine model has allowed for a better understanding of the pathogenic mechanisms in colitis-associated cancer, as it is currently the closest model to this unique scenario. In this review, we provide a summary of the main molecular mechanisms and the clinical aspects of colitis-associated cancer in ulcerative colitis.

## 1. Introduction

Ulcerative colitis (UC), one of the two main forms of inflammatory bowel disease (IBD), is a chronic condition of complex etiology characterized by inflammation limited to mucosa, that extends from the rectum to proximal segments of the colon [1]. The specific cause of IBD has not been established, and currently it is thought to be due to several factors involved in the pathogenesis; some of these factors include molecular and cellular mechanisms, microbial entities, microbiome interactions, genetic anomalies, and immune system disfunction [2]. As with many other inflammatory conditions, a higher risk of malignancies has been established in patients with UC with a cumulative cancer risk of 1.6%, 8.3%, and 18.4% at 10, 20, and 30 years of disease duration, respectively [3]. The risk of colitis-associated cancer (CAC) appears to be related to various factors, such as age at diagnosis, years of disease duration, and extension of colitis. In a Scandinavian population-based study, it was identified that childhood-onset UC, extensive colitis, the co-occurrence of primary sclerosing cholangitis, and a family history of colorectal cancer increased the risk of developing and dying of CAC [4]; in this review, CAC refers exclusively to colon cancer arising from mucosa affected by ulcerative colitis. The aim of this review is to provide a comprehensive review of several molecular mechanisms involved in the development of CAC, the murine models best suited to the study of CAC, as well as the follow-up and management of dysplasia in UC patients.

## 2. Molecular Mechanisms

**Figure 1:**
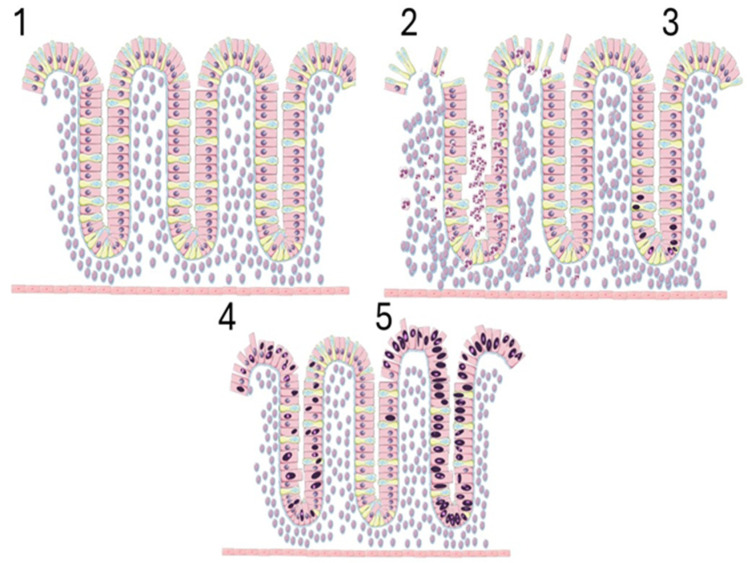
The sequence of CAC tumorigenesis—pink and yellow cells represent enterocytes and goblet cells, respectively; 1.—Normal mucosa; 2.—Active inflammation, with neutrophil infiltrate and inflammatory expansion of lamina propria; 3.—Low-grade dysplasia, and hyperchromasia (represented by dark nuclei), with prominent nucleoli confined to the crypts’ bottom 4.—High-grade dysplasia, abundant aberrant nuclei, hyperchromasia, and abnormal mitotic figures; 5.—In situ carcinoma with multiple aberrant mitotic figures, and extended hyperchromasia with aberrant nuclei.

CAC has different pathways involved in carcinogenesis compared to sporadic colorectal cancer (SCR). While the first follows a sequence of inflammation–dysplasia–adenocarcinoma (as seen in Figure 1), the latter only shows the typical adenoma–adenocarcinoma sequence of colorectal cancer; this is explained by specific genetic anomalies [5]. In SCR, adenomatous polyposis coli (APC) is an early mutation associated with the appearance of adenoma, and Tp53 mutations are related to malignant transformation [6]; on other hand, Tp53 alteration occurs in earlier stages of CAC development [7,8].

### 2.1. P53

Protein p53, a well-known tumoral suppression gene, as mentioned before, is an early altered gene in CAC, mutating before dysplasia can be identified on biopsy specimens [7,8]. Mutations on p53 can have different phenotypes mainly attributed to three mechanisms: loss of function, dominant negative effect, and gain of function. All mechanisms can result in the loss or reversal of anti-tumor pathways [9]. Gain-of-function mutations are thought to be the cause of p53 being identified as an overexpressed protein in some cancers [9]. Most studies evaluating p53 expression are based on immunostaining in biopsy specimens, while identifying the specific mutations is usually carried out by new generation sequencing. In a meta-analysis of 19 studies performed by Yu et al., the expression of p53 on different stages of UC carcinogenesis was evaluated; they found that UC patients had a higher expression of p53 compared to normal controls (OR = 3.14, *p* = 0.001). This was also observed for UC patients with dysplasia vs. UC without dysplasia (OR = 10.76, *p* < 0.001), and, although less significantly, this pattern was also present in UC patients with cancer vs. with dysplasia (OR = 1.69, *p* = 0.035) [10]. As the overexpression of p53 is notably higher in patients with dysplasia, it has been proposed that p53 immunostaining should be performed to differentiate regenerative changes from intraepithelial lesions and dysplasia [11]. In an exome sequencing study conducted by Robles et al., it was reported that the most common mutation in UC–related CRC was Tp53, with a 63% prevalence, similar to those reported on SCR of around 60% [12]. Alterations of Tp53 are often either loss of heterozygosity with a mutation of the remaining allele, occurring in 83% of CAC specimens and 32% of dysplastic lesions, or the mutation of both alleles, found in 32% of dysplasia specimens [7]. The sequencing of the Tp53 gene in aberrant crypts has shown a mutation hotspot in codon 248 (exon 7), where it leads to an amino acid change from Arg to Trp and many other silent mutations occurring in exon 10, with arginine coding codons being described as hotspots in many human cancers [7,12,13,14].

### 2.2. PI3K/AKT

Phosphoinositide-3-kinases (PI3K) belong to an enzyme family characterized by the ability of phosphorylating different phosphoinositides. This family of enzymes has been identified as key messengers related to tumorigenesis, as they possess the ability to promote several cellular responses such as motility, proliferation, and survival, as well as influence vesicle trafficking [15]. Protein Kinase B, historically abbreviated AKT [16], functions as an information link for PI3K [15]. The PI3K/AKT pathway is a regulator of inflammatory responses [17]. This pathway has also been proposed as a therapeutic target in UC for its capability to promote glycolysis in activated T cells, mainly Th2 and Th17, through GLUT4 translocation to the extracellular membrane, which ensures a quick and steady energy source to sustain inflammatory responses [18]. The involvement of PI3K/AKT appears to be due to the cytokines it promotes when activated by other mediators such as TNF alpha; nevertheless, mutations on PIK3CA, the encoding gene of the catalytic subunit of PI3K, have been found in CAC patients [19]. In a study of colonic biopsies from UC and CAC patients, they performed a sequencing of the following 13 cytokine-induced PI3K-related genes: IL12Rß1, IL12Rß2, IL23R, IL31, JAK2, OSMR, STAT1, STAT3, STAT4, STAT6, TYK2, SGK2, and PDK1. The results showed that the most significant variant was in IL23R, rs10889677 (c.*309C>A), where it was present in most of the biopsies from patients with UC and CAC; the second most frequently altered gene was interleukin-12 receptor beta 1, being found in 20% of all samples [20,21]. These mutations have been consistently identified among all biopsies, suggesting that the mechanisms by which they enact their influence on carcinogenesis remain to be precisely characterized.

### 2.3. TLR4/NF-kB

Toll-like receptors are class I transmembrane proteins mostly present in immune cells, involved in the induction of cytokine production and innate immune responses. Upon activation by lipopolysaccharide, toll-like receptor 4 (TLR4) causes the activation of MyD88-dependent and non-MyD88-dependent signaling. MyD88-dependent signaling leads to NF-kB activation that translocates to the nucleus, where it promotes TNF-alpha, IL1, IL6, COX-2, and nitric oxide synthase expression (NOS) [22]. TLR4 has been identified as playing a part in UC pathogenesis, as its inhibition in a DSS-induced colitis mice model demonstrated the amelioration of disease [23]. A mutant variant of TLR4 affecting exon 3 of TLR4, resulting in a change in the amino acid Asp299Gly, has been observed to be present in 14% of patients with UC and has clinical significance in the UC phenotype, involved with specific clinical features such as pancolitis [24]. The mutation Asp299Gly is thought to cause a change in the extracellular domain and alter ligand recognition, as well as enhance inflammatory responses such as a higher TNF alpha levels [25,26]. The expression of TLR4, as well as TNF-alpha and NOS, has been demonstrated to be higher in UC patients, especially in those with CAC compared to healthy controls [27]. Apart from the stablished anti-TNF-alpha therapy, other compounds have been evaluated to alter this pathway, such as artesunate, a derivative of artemisinin, an antimalarial treatment with anti-tumoral and anti-inflammatory properties, which has shown promising results in dextran sodium sulfate-treated mice [28]. All-trans retinoic acid has shown in an in vitro study of CAC and UC cultures to modulate the effects of TLR4/NF-kB and the downregulation of TNF-alpha expression. This effect is achieved by the action of two nuclear receptor families, retinoic x receptor (RXR) and retinoid acid receptor (RAR), which in turn bind to DNA sequences termed retinoic x response elements (RXRE) and retinoic acid response elements (RARE), respectively. In several tumors, such as ovarian adenocarcinoma, breast cancer, and head and neck tumors, retinoic acid has been shown to induce cell cycle arrest in the G1 phase and modulate their growth [27,29,30].

### 2.4. COX-2/PG

Cyclooxygenases are enzymes responsible for the transformation of arachidonic acid to prostaglandin (PG). COX has 2 main isoforms, COX-1 and COX-2. The prostaglandins produced by the inducible COX-2 are mostly involved in inflammatory responses; while both main COX isoforms have been identified to play an active role in the pathogenesis of cancer, COX-2 has been more extensively studied for this role [31]. One of the main products of COX-2 is prostaglandin E2 (PGE2), which can regulate angiogenesis, tumorigenesis, and immunity, as well as promote tumor invasion through PI3K signaling. The mechanisms described are established as relevant mediators in SCR [32]. In UC, COX-2 has been shown to be overexpressed in colonic mucosa [33], but it does not appear to be related to disease phenotype [34]. Expression appears to be higher in patients with UC and dysplasia, as well as CAC, especially in the lamina propria, compared to UC patients without dysplasia, who show no difference in expression when compared to healthy controls [35]. Nonsteroidal anti-inflammatory drugs, inhibitors of COX, have been pointed out as disease activity inducers, especially at high doses [36,37], but recent data are starting to question this effect, as a meta-analysis found no association between NSAID and flares in neither UC nor CD [38]; furthermore, a Cochrane review found no association between COX-2 selective inhibitors and disease relapses. However, these studies have some limitations, such as design flaws, and the follow-up time is short [39]. As COX-2 has been found to be overexpressed in UC and UC with dysplasia, inhibiting this pathway could prevent CAC. For many years, COX inhibitors, represented by NSAIDs, were thought to cause flare and activity in UC, yet new evidence indicates that using COX 2-selective inhibitors might be a safe strategy—more studies are needed to better understand the potential role for preventing CAC.

### 2.5. CA2/CA9

Carbonic anhydrases (CAs) are enzymes well known for their ability to convert carbon dioxide to bicarbonate ions and maintain pH balance [40]. CA 9 is present in colonic mucosa, mostly at the crypts [41], as tumors often develop acidic environments. Due to its demanding metabolism, CA helps maintain homeostasis at the tumor site; in patients with SCR, it has been shown that CA are more present in lymphocytes of healthy mucosa compared to tumor-infiltrating lymphocytes, and this finding outlines the implication of CA in immune disfunction in SCR [42]. Polymorphisms in CA9 have been associated with SCR progression, but the mechanism underlying this association still remains to be evaluated [43]. CAs are implicated in the UC disease course and tumorigenesis, and autoimmune mechanisms against CA have been described in UC patients but not in CD patients [44]. A proteomic and immunoreactivity analysis performed in UC, CAC, and healthy patients showed that CA2 is expressed at a rate 1.5 times lower in UC and CAC patients compared to healthy controls, while CA9 is higher expressed in CAC compared to UC patients; these findings suggest that CAs are involved during the disease course and carcinogenesis, and might be of use as markers for malignant transformation [45]. In a mice model of UC, the administration of human CA1 showed decreased cytokine production and improved symptoms [46]; more studies are required to better characterize the role these enzymes can play in disease management.

### 2.6. Micro RNA

Micro RNAs (MiRNAs) are short non-coding RNAs composed, on average, of 22 nucleotides; they function as key gene expression regulators, as they can silence gene expression and promote RNA degradation, as well as participate in cell–cell communication [47]. MiRNAs have different effects on UC course and CAC development, implicated in both anti-tumoral and pro-tumoral mechanisms by regulating classical disease mediators such as NF-kB, TNFa, iL 1B, and iL6 [48].

#### 2.6.1. MiRNA-193a-3p

This miRNA exerts an anti-tumoral effect by inhibiting IL17D expression in the colonic mucosa of UC patients; miR-193a-3p is significantly reduced compared to healthy mucosa, and even more reduced in malignancy compared to UC tissue samples. Although, paradoxically, IL17D has been found to be overexpressed in cancer samples, some findings suggest it is not exclusively procarcinogenic, as it is thought to have a double effect depending on its exposure to the epidermal growth factor, both in terms of inhibiting cell growth and promoting it [49].

#### 2.6.2. MiRNA -21

This has been extensively studied in other cancers, including colorectal cancer, both as a diagnostic and prognostic tool [50,51,52]. In CAC, it was identified to be overexpressed in human samples, and in MiRNA-21-knockout mice, cytokines showed decreased expression [53], remarking its clear involvement in CAC development; in a UC zebra fish model, it was demonstrated that it promotes CAC by activating the PI3K/AKT, NF-kB/TNF alpha, and JAK/STAT signaling pathways [54].

#### 2.6.3. MiRNA 214

MiRNA 214 has been shown to be able to inhibit NF-kB and improve inflammatory conditions, such as osteoarthritis, as well as inhibit cell proliferation in prostate cancer [55,56]. In UC, it was shown that it was significantly increased compared to healthy controls, and that its expression correlates with disease activity; furthermore, this expression becomes amplified with disease progression to CAC [57].

Although MiRNAs are promising molecules for diagnostic, prognostic, and possibly treatment purposes, further studies are need to better understand what their best use in clinical practice might be.

**Figure 2:**
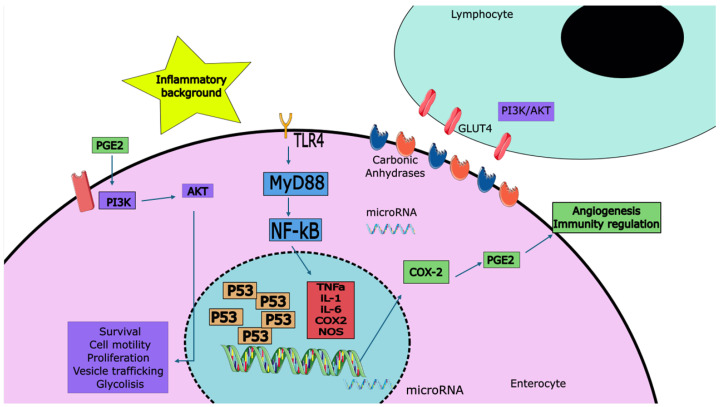
A summary of the molecular mechanisms of CAC. Tp53 mutations cause an overexpression and loss of function. TLR4 alterations, through MyD88-dependent signaling, induce and intensify the proinflammatory environment. COX-2 induces PGE2 expression, which in turn causes angiogenesis and immunity regulation through PI3K signaling. PI3K/AKT is known to promote many cytokines and promote GLUT4 migration to enhance metabolism in TH2 and TH17 lymphocytes. Carbonic anhydrases regulate the acidic environment, associated with high metabolic demands. MiRNA are present in both nuclear and extranuclear spaces, with diverse functions as gene expression regulators.

For a graphic summary of the discussed molecular mechanisms please refer to Figure 2.

## 3. Cellular Mechanisms

### 3.1. Autophagy

Autophagy is a process by which cells break down unneeded intracellular elements and recycle their components. This mechanism is involved in nutrient stress scenarios, when the degradation of components is needed to secure energy sources, and also functions as a housekeeping mechanism, eliminating damaged organelles, misfolded proteins, and other intracellular elements. There are three types of autophagy: chaperone-mediated autophagy, micro-autophagy, and macro-autophagy, with the latter being the focus of this section [58].

In cancer, autophagy was thought to be an anti-tumorigenesis factor, mainly because of its capacity to eliminate damaged elements from the cell, but there is increasing evidence that this mechanism is also involved in tumor progression by aiding in the metabolic shift that cancer cells need to allow their accelerated growth rate [59].

Inflammation during the UC course results in damage to organelles and even in important tumor suppressor genes such as p53; as discussed earlier, the role of autophagy in CAC development appears to be dependent on tumor developmental stage, with its housekeeping role that promotes mucosal healing. Once cancer transformation has occurred, it gains a cytoprotective effect by enhancing the apoptosis and digestion of cells to secure more energy sources for tumoral cells [60]. Autophagy has been studied in murine models, where autophagy was studied in ATG7-deficient and wild-type mice; ATG7 is a core molecule involved in the autophagy process in mammals and in the administration of thiopurine-ameliorated colitis in wild-type but not in ATG7-deficient mice, demonstrating that the effect on inflammation control its due to autophagy. Additionally, a reduction in CAC in both groups was observed, suggesting the existence of other mechanisms independent of the pro-autophagic actions involved in this process [61].

### 3.2. Macrophages in UC and CAC

Macrophages are a crucial part of the innate immune system, arising mainly from the blood-circulating monocytes that differentiate to macrophages when they enter the extravascular space; as their name indicates, their main function is to phagocyte, although they participate in other processes, such as antigen presentation and immunomodulation through cytokines such as TNF alpha, IL-1, IL-6, interferon (INF), IL-10, IL-12, and IL-18 [62]. Subpopulations of macrophages have classically been identified based on whether they are CD14hi CD16 (−), which accounts for “resident” macrophages/monocytes or “inflammatory” macrophages/monocytes which show CD14+ CD16+; however, recently, an “intermediate” group of macrophages has been identified, expressing CD14hi and CD16 (+) [63,64]. The activated macrophages follow a process termed polarization, by which they acquire certain properties and activities; inflammatory macrophages are normally activated by TNF alpha, INF, and lipopolysaccharide products as M1 macrophages that have increased antigen-presenting capacities and secrete various proinflammatory cytokines such as TNF alpha, IL-1, IL-6, and IL12, among others, resulting in anti-tumoral, anti-microbial, and proinflammatory effects, while resident macrophages are activated by the Th2 cytokines IL-10 and IL–4, as M2 that display a strong phagocytic capability and promote angiogenesis, tissue repair, and the suppression of immune response [65].

M1 and M2 effects have different impacts depending on the disease course and whether mutations on key genes as Tp53 have already occurred. During the UC course, it is desirable to control inflammation, i.e., to suppress M1 activity to ameliorate disease symptoms and induce remission, while enhancing M2 activity to achieve mucosal healing. Once dysplasia and mutations are already present, M2 immune suppression is not entirely desirable [66].

### 3.3. Neutrophils in UC

Neutrophils represent about 50–70% of all circulating leukocytes in humans and constitutes the most abundant immune cell, which is derived from the myeloid line in the bone marrow and plays an important role in acute inflammation and adaptative immune activation by secreting chemokines (IL1, IL6 and TNFa) and reactive oxygen species (ROS) [67]. Neutrophils keep pathogens at bay and can also contribute to mucosal damage due to excessive activation and recruitment [68]. Their ability to produce ROS links them to carcinogenesis, as these molecules are known mutagens for their capacity to induce genomic damage through C > A mutations and base pair deletions [69,70,71]. An in vitro study of *H*_2_*O*_2_ derived from activated neutrophils also demonstrated their ability produce frame-shift mutations and microsatellite instability, which also contribute to genomic alterations and tumorigenesis [72].

Neutrophil extracellular trap (NET) formation is another complex mechanism that occurs in UC patients. NETs are mainly formed by a special cell death mechanism called NETosis. This process is triggered by several stimuli such as pathogens, immune cells and complexes, crystals producing NADPH oxidase, MPO, and neutrophil elastase (NE) activation, which migrate to the nucleus where chromatin is decondensed [73]. Peptidyl arginine deiminase type 4 (PAD4) is another key molecule for NETosis, as it is capable of histone citrullination, further contributing to chromatin decondensation [73]. Once released from the cell, NETs carry proteases that can damage tissues, as well as citrullinated proteins, which are known to be immunogenic [74]. In UC, PAD4, MPO, and NE have been shown to be significantly higher in colonic mucosa when compared to healthy controls, CDs, and even unaffected mucosa from the same patient; thus, NETs play a role in the inflammation process in UC patients [75]. NETs might have an important role in UC, as well as in the carcinogenesis process, which remains to be defined in future studies.

### 3.4. T Regulator Cells

T regulatory cells, or Tregs, are a class of lymphocytes dedicated to limiting and regulating inflammatory responses in order to prevent excessive activation and self-damage. Tregs express FOXP3 as the most specific marker, and it has been shown that FOXP3-deficient individuals have severe autoimmune/autoinflammatory conditions, like immune dysregulation, polyendocrinopathy, enteropathy, and X-linked syndrome (IPEX) [76,77]. Treg cells can infiltrate tumors such as melanoma by expressing the C-C chemokine receptor type 4 (CCR4), and depleting Tregs can effectively produce anti-tumor immune responses [78]. Some studies suggest that Treg cells have plasticity and can transform into Th17 cells and enact a proinflammatory effect [79]. The role of Tregs in UC patients appears to be to produce IL-1 and IL-6, acting as a special effector cell in UC and CAC conditions [80].

## 4. Animal Models for CAC Study

By far the most common animal model in CAC is the azoxymethane/dextran sodium sulfate murine model, a chemically induced colitis model. Azoxymethane (AOM) is a procarcinogen that is metabolized by the liver to methylazoxymethanol, its active form, which gets secreted through bile into the digestive tract of the mouse; the administration of AOM is followed by a strong inflammatory insult, using dextran sodium sulfate (DSS) [81]. This combination generates severe bloody diarrhea in mice that leads to weight loss and, with multiple cycles of DSS, to tumorigenesis in various sites in the mice colon [82]. This model has enabled the study of several aspects of CAC molecular pathways and mutations in a fast-paced manner, as it is quick to show cancer development; though not a perfect model, it offers a great platform for understanding novel mechanisms and proposing treatment targets.

Spontaneous models are almost never used, as they are unpredictable and have low reproducibility; even in cancer prone mice strains, the incidence of colon carcinoma goes from 40 to 60%. Genetic engineered models have used mice with classically cancer associated genes artificially altered; however, they work better to understand the hereditary syndromes of colon cancer such as familial adenomatous polyposis. Transplant models are based on taking tumor cells from a mouse and implanting them on another; these allow us to understand cancer invasion mechanism, but lack value as pathogenesis models [83,84].

CAC rises from the inflammation–dysplasia–carcinoma sequence, and as such, the chemically induced cancer models provide the nearest approximation to the fundamental events that occur in the progression from UC to CAC; apart from the AOM/DSS model, other chemical compounds have been used to induce cancer in murine models such as 3,2′-Dimethyl-4-Aminobiphenyl (DMAB), N-Methyl-N-Nitrosourea (MNU), 1,2-Dimethylhydrazine (DMH) [84], yet they lack the background inflammation needed to emulate CAC, as they cause direct DNA damage.

The DSS/AOM model is not truly standardized, and many studies have slightly different methodologies; as such, the reproducibility of the experiments is questionable. A systematic review performed by Modesto et al. found that the DSS effect on inflammation was dose dependent, with a 3% DSS solution added in water being the most used; repeated exposures were sometimes used to emulate chronical inflammation, while the AOM dose most frequently used was 10 mg/kg in a single dose to induce carcinogenesis, and the most frequently used mice strain for CAC is C57BL/6, followed by Balb/c mice [85]. Another proinflammatory agent suggested for CAC is y 2,4,6-trinitro-benzene sulfonic acid (TNBS); however, as it causes transmural inflammation, it resembles CD rather than UC and as such, cannot be used as a true model for UC-related cancer [86].

## 5. Dysplasia and Cancer Screening in Ulcerative Colitis

### 5.1. Types of Dysplasia

Dysplasia’s endoscopic nomenclature and reporting are currently based on the modified Paris classification in accordance with AGA’s current guidelines, using descriptors such as polypoid (>2.5 mm tall), including pedunculated and sessile polyps, non-polypoid, (<2.5 mm tall) described as flat, flat—elevated, and flat—depressed, and a unique dysplastic lesion found by random biopsies named invisible dysplasia [87]; according to expert recommendations, older nomenclature should be avoided.

At the histologic level, dysplasia is described using the 1983 Ridell and Colleagues system (Table 1), dividing the morphologic characteristics into four grades: normal, indefinite for dysplasia, and low-grade and high-grade dysplasia [88,89]. Although the classification is widely used all over the world, interobserver variation has been reported; in a study conducted by Leoncini et al., they reported an interobserver agreement in the presence of dysplasia of 72.3%, and when evaluating the grade of dysplasia agreement, they found an agreement of 65%, highlighting the challenge of dysplasia recognition at the histological level even when evaluated by expert gastrointestinal pathologists [90].

Low-grade dysplasia has a prognostic implication, because it may evolve into high-grade dysplasia or CAC. A meta-analysis reported a nine-fold increase in risk of developing cancer when low-grade dysplasia was diagnosed, and a twelve-fold increased risk of developing an advanced lesion [91]. Other predictors for developing high-grade dysplasia include non-polypoid or invisible lesions, large (>1 cm), or those preceded by indefinite dysplasia [92].

### 5.2. Cancer Screening in Ulcerative Colitis

In SCR, there are a variety of approved screening tests, such as fecal immunochemical test (FIT), guaiac test, computed tomographic colonography, sigmoidoscopy, and colonoscopy [93]. For CAC, the most used screening tool is colonoscopy, as it provides both disease and dysplasia surveillance. Evidence about the effectiveness of colonoscopy screening for improving survival is limited, because there are no randomized clinical trials available on this matter; a Cochrane review that included 4 cohort studies and 1 case–control study concluded that patients that were screened for CAC were diagnosed in earlier stages, 9% of patients in surveillance had late-stage cancer compared to 16% of patients in non-surveillance (OR = 0.46, CI 95%: 0.08 to 2.51, *p* = 0.37), and the mortality associated with CAC was higher in non-surveillance vs. surveillance patients, 22.3% vs. 8.5% (OR = 0.36, CI 95%: 0.19 to 0.69, *p* = 0.002). As mentioned in the study, the quality of the evidence is low, as the study designs are not adequate [94]; similar findings have been more recently reported in a registry-based study performed by Narula et al. [95].

**Figure 3:**
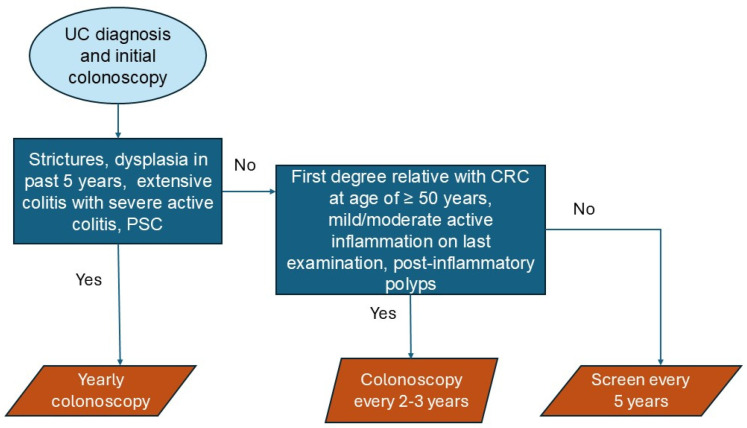
CAC screening frequency algorithm.

Surveillance colonoscopies are usually started after 8–10 years of diagnosis or symptom onset of UC in accordance with the latest ACG and ECCO guidelines, respectively; surveillance schedules should be individualized, taking into account risk factors for developing colorectal cancer—high-risk features include strictures, dysplasia detected in past 5 years, extensive colitis with severe active inflammation, and concomitant diagnosis of primary sclerosing cholangitis (PSC). The presence of any of these characteristics constitute a high risk, and screening should be scheduled annually. Intermediate risk patients should be screened every 2–3 years—intermediate risk includes those with a first-degree relative diagnosed with CRC at age of ≥50 years, mild or moderate active inflammation on last examination, or with post-inflammatory polyps; if neither high nor intermediate risk features are present, screening is recommended every 5 years [87,96,97], as shown in Figure 3.

Several colonoscopy imaging modalities have been evaluated to detect dysplasia, including high-definition white light (HD-WL), standard-definition white light (SD-WL), dye-based chromoendoscopy (CE), and narrow-band imaging (NBI). In a recent meta-analysis performed by Gondal et al., they evaluated the efficacy of dysplasia detection per biopsy analyzed and per patient; in the biopsy analysis, they found that both NBI and CE were more efficient than WL modalities, while in the patient analysis they concluded that WL-SD was inferior to the other three modalities [98].

Biopsies are an essential tool in UC, as they provide both histologic activity evaluation and dysplasia/CAC detection; four quadrant random biopsies every 10 cm have been recommended as a method for biopsy collection, but as it is time- and resource consuming, it has been criticized, and, with the development of the different imaging modalities previously discussed, targeted biopsies have become a good alternative strategy. A randomized multicenter clinical trial performed by Wan et al. evaluated the dysplasia detection rate for white light with targeted biopsies (WLT), white light with random biopsies (WLR), and CE with targeted biopsies (CET); the results showed that both CET and WLR detected more dysplastic lesions than WLT (10.7%, 9.3% and 2.5%, respectively, *p* = 0.014), and the number of biopsies taken, as expected, was significantly higher for the WLR arm, with a mean of 16.5 compared to a mean of 4.4 in both WLT and CET. From these results, we can conclude that although a similar amount of dysplastic lesions are identified with both CET and WLR methods, CET needs less biopsies to detect the same amount of lesions, resulting in both less resources and time expended [99].

Colonoscopy remains the best tool for CAC screening, yet better quality studies are needed to truly understand the impact on survival and dysplasia detection; of the modalities employed during colonoscopy, the best methods appear to be CET and NBI.

### 5.3. Dysplasia Management

Once dysplasia has been identified and characterized, the therapeutic approach should be carefully selected, considering whether endoscopic management is feasible and accessible or if surgical intervention is needed. For polypoid lesions, endoscopic resection during colonoscopy is recommended, as it is low risk, and no requires special skills, but if lesions are complex or flat, advanced methods such as endoscopic submucosal dissection or endoscopic mucosal resection are needed before considering colectomy [87,96]. Endoscopic submucosal dissection is a safe and effective method for dysplasia management. A meta-analysis reported a complete resection of 84% and a curative rate of 81%, and a low rate of complications, finding a pooled incidence of bleeding, perforation, and recurrence of 8%, 6%, and 5%, respectively, with similar results to the case series and other studies [100,101,102,103]. These findings support the current strategy of resect-and-observe. Surgical approaches are reserved for those patients in whom endoscopic resection is not possible, or if there is a lack of materials and professionals.

## 6. Chemoprevention

Cancer chemoprevention is defined as a pharmacological intervention in order to reverse or stop the carcinogenesis process [104]; as has been previously discussed, CAC follows a unique pathogenesis within colorectal cancer, focusing on chronic inflammation and cumulative genomic damage to colonic epithelial cells, and as such, chemoprevention is based mainly on inflammation control and an induction of remission, where several chemopreventive/therapeutic agents have been evaluated.

### 6.1. 5-Aminosalicylic Acid Compounds (5-ASA)

5-ASA compounds remain one of the main therapeutic options for patients with IBD, especially for UC patients, being widely available and recommended in current guidelines for both the induction and maintenance of remission [96,105,106], as well as chemoprevention [97,106], since its first report as a chemopreventive in UC 30 years ago [107]. A meta-analysis performed by Qiu et al. explored the effects of 5-ASA therapy as a chemoprevention for both cancer and dysplasia in UC, finding in the pooled analysis that 5-ASA therapy had a chemopreventive effect in the combined outcome of CAC/dysplasia with an OR = 0.58 (95% CI: 0.45–0.75, *p* = 0.000); however, in UC, the sub-analysis found that this effect was only present for the CAC outcome (OR = 0.40 95% CI: 0.30–0.55,) and no protective effect for dysplasia (OR = 0.18, 95% CI: 0.02–1.53) nor CAC/dysplasia outcomes (OR = 0.62, 95% CI: 0.36–1.06) was observed; they also evaluated if this effect was dose dependent, finding that doses of >1.2 g of mesalazine had a chemopreventive effect and sulfasalazine effect was not dose dependent [108].

### 6.2. Thiopurines

Thiopurines are currently recommended as a therapy for patients with steroid dependence or as an adjunct to anti-TNF alpha blockers, especially with infliximab [96,105], though its use as a chemopreventive is not widely accepted [96,106]; conflicting evidence regarding its protective effect has been published [109,110,111,112,113], but increasing evidence showing a low to no effect on CAC/dysplasia development prevention has been the norm, with a recent trend indicating a chemopreventive effect among case–control studies but not in population-based studies. More studies with higher impact are needed to better understand the effect of thiopurines on carcinogenesis, as the bulk of the evidence comes from cohort studies.

### 6.3. Anti-TNF Alpha Blockers

Biologic and small molecule therapy represent the modern era of IBD treatment, as they impact on specific pathophysiological mechanisms of disease perpetuation. Tumoral necrosis factor alpha, as previously discussed, is involved in UC carcinogenesis; as such, therapy with these agents provides an induction of remission, maintenance, and a theorical chemopreventive effect through the blocking of TNF alpha and its resulting/dependent pathways, such as TLR4-MyD, PI3K, MiRNA-21, and M1 macrophages. A French nationwide cohort study showed that exposure to anti-TNF alpha agents such as infliximab, golimumab, adalimumab, and certolizumab decreased CAC risk, especially in those with prolonged disease duration (>10 years), with an HR = 0.41; (95% CI: 0.20–0.86) [114]. In a multicenter study conducted in the US, they reported that concomitant use of immunomodulators showed that anti-TNF alpha reduced the risk of CAC (OR = 0.78; 95% CI: 0.73–0.83; *p* < 0.0001) and the concomitant use of immunomodulators also had a protective effect (OR = 0.83; 95% CI: 0.70–0.99; *p* = 0.0378). This study also evaluated the effect of other biologic therapies, including ustekinumab, vedolizumab, tofacitinib, and natalizumab, and none of them showed an effect on the prevention of developing CAC [115].

## 7. Conclusions

CAC represents a multilayer and complex problem. We described a series of alterations ranging from receptors, such as the toll-like receptor 4, to core genetic alterations such as those that occur in p53; these alterations lead not only to genomic instability but also pro-tumoral microenvironments, as is the case with carbonic anhydrases. Many breakthroughs have been made thanks to the DSS/AOM model, and currently it is the most used model for its reliability, and the relatively fast onset of colorectal cancer. Trying to separate the pathogenesis and perpetuation conditions of ulcerative colitis from CAC is not yet possible, as current understanding points out that cancer in this scenario should not be thought of only as a possible complication but as an end stage of uncontrolled disease and cumulative damage. More studies dedicated to understanding the unique pathogenesis of this entity are needed to direct future efforts towards a greater understanding and better care for patients afflicted by IBD and their associated cancers.

## Figures and Tables

**Table 1:** Types of dysplasia in UC.

Dysplasia Grade	Histological Findings
Negative for dysplasia	Normal appearance in biopsy specimens, or showing reactive changes to inflammation (nuclear atypia adjacent to ulceration, mildly hyperchromatic nuclei, etc.).
Indefinite for dysplasia	Specimens in which it is not possible to differentiate reactive from dysplastic changes or with dysplasia features in a non-sufficient sample.
Low-grade dysplasia	Preserved cellular polarity, nuclei confined to basal half of cells, typical mitotic figures, uniform nuclear size and shape, inconspicuous nucleoli, relatively low nuclear to cytoplasmic ratios.
High-grade dysplasia	Loss of nuclear polarity, nuclear stratification involving luminal half of cells, high nuclear to cytoplasmic ratios, markedly enlarged nucleoli, atypical mitotic figures, marked nuclear hyperchromasia and pleomorphism, intraluminal necrotic debris, complex architecture (fused or incomplete tubules, cribiform).

## Data Availability

No new data were created or analyzed in this study. Data sharing is not applicable to this article.
